# Establishing hazelnut stem water potential baseline to improve water management

**DOI:** 10.3389/fpls.2026.1771736

**Published:** 2026-03-27

**Authors:** Giacomo Dito, Sofia Russo, Nik G. Wiman, Sergio Tombesi

**Affiliations:** 1 Department of Sustainable Crop Production, Università Cattolica del Sacro Cuore, Piacenza, Italy; 2 North Willamette Research and Extension Center, Oregon State University, Aurora, OR, United States

**Keywords:** filbert, hazelnut tree, irrigation, over-irrigation, stem water potential baseline, water management

## Abstract

**Introduction:**

Irrigation management in modern filbert (*Corylus avellana* L.) orchards is becoming increasingly important due to climate change, with rising temperatures and more frequent arid periods occurring across many hazelnut growing regions. While irrigation is essential to sustain productivity, water scarcity in several areas highlights the need for efficient and sustainable irrigation strategies. Stem water potential (SWP) is one of the most reliable indicators for assessing plant water status, yet no species-specific baseline has been defined for filbert under non-limiting soil moisture conditions. This study aimed to establish an SWP baseline for filbert, following approaches previously developed for olive and *Prunus* species.

**Material and Methods:**

Vapor pressure deficit (VPD) was used as the environmental driver defining SWP variability under well-watered conditions. A large dataset of SWP–VPD pairs was collected during the 2024 growing season in two major hazelnut-producing countries, Italy (Piacenza) and Oregon (USA), and supplemented with measurements from previous trials.

**Results and discussion:**

The resulting baseline, derived from the upper 0.07 fraction of SWP values within VPD classes, revealed a stable and linear relationship between SWP and VPD. This baseline functions as an operational reference: SWP values close to the baseline indicate non-limiting soil water conditions, negative deviations reflect increasing water stress, whereas values exceeding the baseline may signal excessive soil moisture. Overall, the proposed SWP baseline provides a plant-based threshold that can be directly applied in field irrigation management to optimize timing and amount of water application and can be integrated with other plant-based indicators, such as baseline deviations or the Crop Water Stress Index (CWSI), within threshold-based or advanced irrigation decision-support frameworks, supporting more sustainable water use under increasingly variable climatic conditions.

## 1 Introduction

In modern horticulture, it has been proven that irrigation provides a number of benefits. Hazelnut trees orchards are historically not irrigated, but in recent years, with the increase of temperature in summer, irrigation systems have been commonly adopted all over the world. Irrigation significantly influences the physiological processes of hazelnut trees (*Corylus avellana* L.), impacting photosynthesis, water relations, and overall plant health. Adequate water supply enhances leaf gas exchange parameters, including net photosynthesis rate (*A*_n_), stomatal conductance (*g*_s_), and internal CO_2_ concentration (*C*_i_) (Tombesi, 1994). This improvement supports better growth and productivity. Conversely, water stress can lead to reduced chlorophyll content and lower levels of photosynthetic pigments, adversely affecting plant vitality (Dias et al., 2005). Past studies have shown that irrigation strongly improves hazelnut growth and yield compared with non-irrigated conditions; however, increasing irrigation beyond crop evapotranspiration requirements does not result in further yield benefits. For example, Girona et al. (1994) reported clear differences between irrigated and non-irrigated treatments, but only marginal or negligible yield responses when irrigation exceeded optimal levels. In addition to the lack of productive advantages, excessive irrigation in hazelnut orchards has been associated with agronomic and physiological risks, particularly in soils with limited drainage capacity. Rowe and Beardsell (1973) reported that waterlogging in fruit trees reduces soil oxygen availability and impairs root function, leading to reduced growth and physiological performance. Experimental evidence in woody species further indicates that tolerance to waterlogging is closely linked to the capacity for internal oxygen transport from aerial tissues to submerged roots, a mechanism that is poorly developed in flood-intolerant species (Philipson and Coutts, 1978). These findings support the concept that excessive irrigation may promote transient root-zone waterlogging and oxygen limitation in tree crops. In the context of climate change, where water availability is expected to become increasingly constrained during the hottest periods of the growing season, improving irrigation management strategies is therefore essential not only to avoid water deficits, but also to prevent the negative consequences associated with excessive water application (Sabir, 2024). To know when over-irrigation occurs, it is necessary to understand what are the thresholds and when the water provided by the irrigation is not a limiting factor for the physiological processes of the plant. To determine the water status of the plant, midday stem water potential (SWP) is a standard tool for plant-based irrigation management in many woody perennial crops (Shackel et al., 2021). In a well-watered system, the only variable factors that can modify SWP during the vegetative season are atmospheric features like temperature and humidity, which can be evaluated by the vapor pressure deficit (VPD) (Shakel et al., 2021). In addition, as for SWP, stomata conductance responds to different VPD conditions, affecting water use efficiency and carbon assimilation (Ocheltree et al., 2014). Cincera et al. (2019) documented intra-specific variability in stomatal sensitivity to VPD. The study showed that different genotypes of *C. avellana* respond differently to different VPD conditions, probably caused by genetic differences. For this reason, data were collected on different filbert varieties across multiple regions worldwide.

The experiment hypothesis is that SWP under non-limiting water availability in the soil is defined by VPD. The SWP–VPD relationship defines the maximum SWP that can be reached at specific weather conditions.

The aim of this study is to create a hazelnut SWP baseline to prevent excess irrigation.

## 2 Materials and methods

To develop a robust SWP baseline representative of non-limiting soil water conditions across a wide range of atmospheric demand, data were collected from hazelnut orchards differing in climate, cultivar, and irrigation management. The inclusion of geographically distinct sites was intentionally aimed at expanding the variability of VPD conditions under which SWP was measured, while ensuring that all measurements were conducted on fully irrigated trees. As the SWP baseline is defined as the upper limit of the SWP–VPD relationship under non-water-limiting conditions, site-specific differences in climate and management were considered functional to baseline construction rather than confounding factors. The experiment was carried out in Piacenza, Italy and Corvallis, Oregon.

### 2.1 Experimental design

#### 2.1.1 Irrigation management and definition of non-limiting soil water conditions

To ensure non-limiting soil water conditions, irrigation was scheduled to fully replace crop evapotranspiration (ET_c_), calculated on a daily basis assuming non-limiting soil water availability. Actual evapotranspiration was estimated as ET_c_ = ET_0_ × *K*_c_ × *K*_r_, where reference evapotranspiration (ET_0_) was calculated using the FAO Penman–Monteith equation (Allen et al., 1998). The crop coefficient (*K*_c_) was set to 0.8 throughout the growing season (April–October), according to lysimetric measurements on hazelnut (Mingeau and Rousseau, 1994), while the reduction coefficient (*K*_r_) accounted for changes in effective ground cover following FAO-56 guidelines. *K*_r_ was calculated as a function of canopy ground cover fraction (*f*_c_), which was estimated geometrically from canopy projection on the soil and progressively increased with tree growth. Irrigation volumes were intentionally set to meet or slightly exceed estimated ET_c_, in order to avoid transient soil water deficits. Under these conditions, irrigation management was used as an operational criterion to define non-limiting soil water conditions for SWP baseline determination.

#### 2.1.2 Piacenza, Italy

The experiment was carried out at the experimental hazelnut orchard of the Università Cattolica del Sacro Cuore (45°02′03.2″N, 9°43′51.7″E; 61 m a.s.l.) in 2024. The orchard (cv Tonda di Giffoni) was drip irrigated. Piacenza is in the south-central part of the Po Valley where the climate features are between the Mediterranean climate and the continental/oceanic one of Central and Western Europe (Nistor, 2016). In 2024, between bud break and leaf senescence (from April to November), the daily average temperature varied between 10 °C and 27 °C with a cumulative annual precipitation of 1,200 mm. The soil was silty clay loam texture based on USDA soil classification (20% sand, 43% silt, and 37% clay). Hazelnut trees were irrigated daily, with irrigation volumes calculated to fully replace or slightly exceed crop evapotranspiration (ETc), in order to avoid soil water limitation throughout the measurement period. The cultivar used was Tonda di Giffoni; half of the plants used for taking measurements were self-rooted, and the other half were grafted on *Corylus colurna*. The measurement period lasts from May to October. The measurements were conducted weekly between 12:00 and 14:00 local time; before taking the measurement, in order to equilibrate the leaf to SWP, leaves close to main scaffolds were wrapped in an aluminum foil that prevented leaf transpiration. After 30 min, leaves were taken from the tree and measured with a Scholander chamber (model 3005H07G4P40, Soil Moisture Equipment, Santa Barbara, CA, USA).

For gas exchange measurements, fully mature and healthy leaves located in the middle of the current-year shoot were sampled. Stomatal conductance (*g*_s_), leaf transpiration (*E*), and net photosynthesis (*A*_n_) were measured during the leaf equilibration period using a portable gas analyzer (LCpro T, ADC BioScientific, Hoddeston, UK) on fully expanded, healthy leaves located in close proximity to the wrapped leaf, with similar age, canopy position, and light exposure. All measurements were performed following the manufacturer’s calibration procedures prior to each measurement campaign. Gas exchange data were used to support physiological interpretation under non-limiting soil water conditions and were not intended for direct quantitative comparison across sites.

#### 2.1.3 Corvallis, Oregon

Experiments were carried out in different commercial hazelnut orchards in the Willamette Valley(44°26′46.9″N, 123°18′15.6″W; 44°26′08.3″N, 123°13′26.6″W), near the city of Corvallis, in Benton County, Oregon (USA), between May and August 2024. The Willamette valley area is characterized by Mediterranean-type climate with warm, dry summers and mild, but wet winters. The mean annual temperature is approximately 10°C to 13°C and receives consistent winter precipitation due to the westerly flow of Pacific storms. The mean annual precipitation is 1,228 mm, ranging from 900 to 1,600 mm in the mountainous foothills (Wiken et al., 2011). The commercial orchards taken in examination were planted with McDonald and Jefferson varieties both at the fifth leaf. In each orchard, trees were trained on a single stem and planted 6 m between rows and 3 m on the row, with a density of 555 trees per hectare and with a North–South row orientation. The irrigation was applied from June to August, with a sub-irrigation system with drippers 60 cm spaced with a flow rate of 2 L per hour. The irrigation shifts lasted 12 h in June and 24 h in July and August, every day. Irrigation scheduling and duration were designed to ensure full restitution of estimated crop evapotranspiration (ETc), thereby maintaining non-limiting soil water conditions during all SWP measurements. Considering tree size, the water volume applied per day was between 140 and 280 L depending on irrigation duration. All SWP measurements were conducted within the same midday time window (12:00–14:00 local time) and following the same leaf selection and equilibration protocol adopted at the Italian site. The same Scholander-type pressure chamber model was used for all measurements (model 3005H07G4P40, Soil Moisture Equipment, Santa Barbara, CA, USA). During the leaf equilibration period, gas-exchange measurements (*g*_s_ and *E*) were taken during the leaf equilibration period using a porometer (LI-600, LI-COR Biosciences, Lincoln, NE, USA) on fully expanded, healthy leaves located in the middle of the current-year shoot. All measurements were performed following the manufacturer’s calibration procedures prior to each measurement campaign. Gas exchange data were used to support physiological interpretation under non-limiting soil water conditions and were not intended for direct quantitative comparison across sites.

### 2.2 Baseline regression

The baseline relationship between midday SWP and VPD was calculated following the method described by Shackel et al. (2021). The full dataset of SWP–VPD pairs was divided into 0.5-kPa VPD classes. Within each class, the upper 0.07 fraction of SWP values (i.e., the least negative) were selected, assuming they represent non-soil-water-limited conditions. Average SWP and VPD values from these subsets were used to perform linear regression. The choice of the 0.07 fraction was based on a sensitivity analysis reported by Shackel et al. (2021), in which upper SWP fractions ranging from 0.02 to 0.16 were evaluated. Among the tested fractions, 0.07 yielded the highest *R*^2^ and the lowest *p*-value, and was therefore considered the most robust and appropriate estimate of the SWP baseline under non-soil-water-limiting conditions. To enrich the dataset, additional measurements from previous trials conducted in irrigated and rain-fed orchards of the cultivars ‘McDonald’ and ‘Jefferson’ were included.

## 3 Results

### 3.1 Midday stem water potential baseline

**Figure 1:**
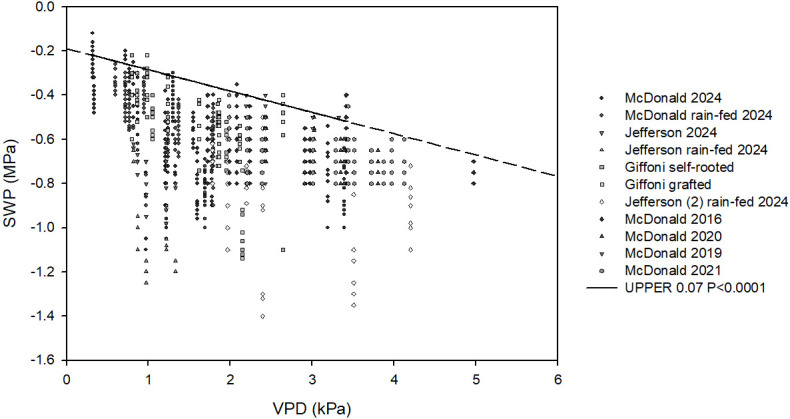
All measured data of SWP (MPa) to midday VPD (kPa) of different varieties in the two selected sites. Each point is an individual measurement, with vertical lines of points indicating SWP values that were collected at the same site and time. The solid black line denotes the regression of the upper 0.07 fraction of the datasets ( SWP (MPa)=−0.09×VPD−0.18   *R*^2^ = 0.80, *p* < 0.0001); the dotted line represents the projection of the rest of the baseline.


**Figure 1** shows the negative relation between SWP and VPD. The dataset used to obtain the baseline had a VPD range from 0.2 to 5 kPa, which was quite similar for both locations. Regarding SWP, the range was from −0.05 to −1.4 MPa, including all varieties irrigated and rain-fed. The upper 0.07 fraction used for calculating baseline regression had a VPD range between 0.2 and 3.5 kPa and an SWP range between −0.22 and 0.55 MPa. The dataset included well-watered and rain-fed trees of different varieties. The regression of the upper 0.07 fraction was linear and had the following equation:

SWP (MPa)=−0.09×VPD−0.18 

*R*^2^ = 0.80 and a significance *p* < 0.0001.

In **Figure 1**, a dotted line was added to represent the extension of the baseline regression.

### 3.2 Leaf transpiration vs. VPD

**Figure 2:**
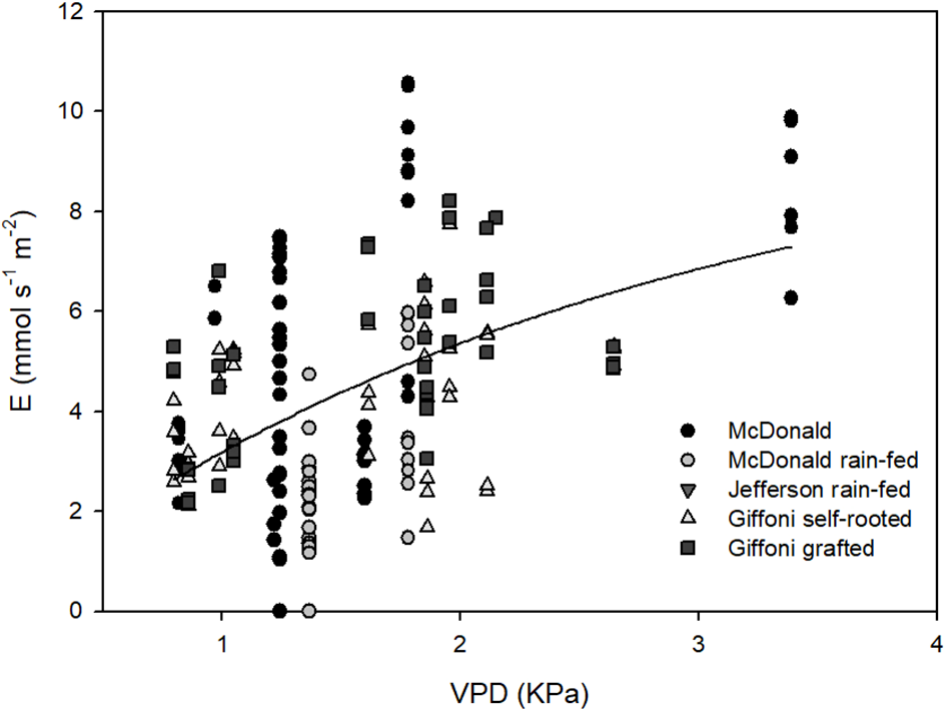
Relation between *E* (mmol m^−2^ s^−1^) and VPD (kPa). Each point is an individual measurement, with vertical lines of points indicating *E* values that were collected at the same site and time. Nonlinear regression results correlated for *p* < 0.0001 (ANOVA).

The relationship between E and VPD (**Figure 2**) was positive, with a steeper increase between 0.5 and 2 kPa, followed by a tendency to flatten at higher VPD values. For this graph, only data taken during 2024 season from ‘McDonald’, ‘Jefferson’, and ‘Tonda di Giffoni’ were used, when uniform and non-limiting irrigation ensured homogeneous field conditions across cultivars. The variability depended on varieties and water source with maximum values obtained in well-watered conditions. The highest *E* value observed was 11 mmol s^−1^ m^−2^ and the lowest was 2 mmol s^−1^ m^−2^.

### 3.3 *g*_s_ vs. SWP

**Figure 3:**
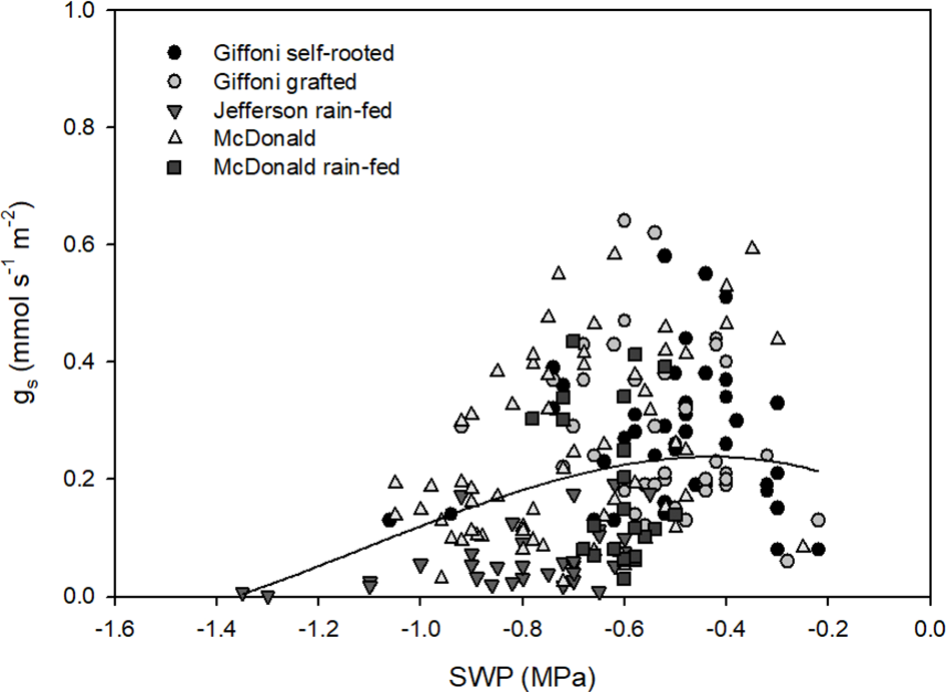
Relation of *g*_s_ (mmol m^−2^ s^−1^) and SWP (MPa). Each point is an individual measurement. The solid line represents the third-degree (cubic) polynomial regression ( $g_{s}\left(mmol\;s^{-1}m^{-2}\right)=0.12-0.05\times SWP-0.008\times SWP^{2}-0.0002\times SWP^{3}\;$ *R*^2^ = 0.06, *p* = 0.0018).

The relation was not linear and tended to saturate after −0.8 MPa, which was the flex point, as shown in **Figure 3**. At −1.4 MPa, *g*_s_ was close to complete stomata closure; from this point, it increased, reaching its maximum at −0.45 MPa after this point, and the trend showed a slight decrease until −0.2 MPa. As for **Figure 2**, the dataset used concerned only data acquired in 2024. Also, in this case, the variability per variety and source of water was high, but looking at the single treatment, trends seemed to be consistent with the regression of the whole dataset. Well-watered orchards (McDonald and Tonda di Giffoni) reached higher values between 0.55 and 0.65 mmol s^−1^ m^−2^ at approximately −0.6 MPa.

## 4 Discussion

### 4.1 SWP baseline definition and response to atmospheric demand in hazelnut

The main objective of this experiment was to establish a reference relationship between midday SWP and atmospheric demand under non-limiting soil water conditions, providing a practical tool for irrigation management in hazelnut orchards and helping to avoid over-irrigation. Following the conceptual framework proposed by Shackel et al. (2021), the SWP baseline represents plant water status under adequate soil moisture availability rather than a non-stressed physiological condition. This distinction is particularly relevant for hazelnut, as similar SWP values may occur either under high VPD and well-watered soil or under low VPD combined with soil water limitation.

In this context, SWP values close to the baseline indicate that irrigation is not limiting plant water uptake and transpiration, whereas deviations below the baseline reflect increasing soil or hydraulic limitations. Conversely, SWP values exceeding the baseline may suggest excessive soil moisture, potentially leading to transient hypoxic conditions and impaired root functioning.

In the present study, the SWP–VPD baseline relationship in hazelnut was linear, with a slope of −0.09 and an intercept of −0.18 MPa. These values fall within the range reported for other woody crops, such as olive (−0.18, −0.34 MPa; Shackel et al., 2021) and *Prunus* spp. (−0.12, −0.41 MPa; McCutchan and Shackel, 1992). Compared with these species, hazelnut exhibited a less steep baseline slope, indicating a smaller decline in SWP per unit increase in atmospheric demand under non-limiting soil water conditions. This suggests a relatively buffered SWP response to increasing VPD when soil water supply is adequate.

Importantly, the slope of the SWP baseline should not be interpreted as a direct indicator of drought tolerance or isohydric behavior. In hazelnut, conservative water-use strategies and high sensitivity to water stress are primarily reflected in the rapid reduction of stomatal conductance and the narrow functional range of SWP observed under declining soil water availability. As shown by the *g*_s_–SWP relationship, stomatal closure occurs at relatively high SWP values compared with more anisohydric species, supporting the classification of hazelnut as an isohydric species.

Overall, the SWP baseline defined in this study provides a robust physiological reference for distinguishing atmospheric from soil-driven reductions in plant water status and represents a valuable component for the integration of plant-based indicators into irrigation decision-making frameworks for hazelnut.

### 4.2 Inclusion of irrigated and rain-fed data in baseline construction

The SWP–VPD relationship presented in this study includes measurements collected under both irrigated and rain-fed conditions, as data were intentionally pooled to cover a wide range of plant water status and atmospheric demand. However, the baseline was not derived from the entire dataset, but was calculated following the upper limit approach proposed by Shackel et al. (2021). By dividing the dataset into VPD classes and selecting the upper 0.07 fraction of SWP values within each class, the analysis isolated measurements representative of non-soil-water-limited conditions, independently of irrigation regime, site, or year.

Under this framework, measurements collected under rain-fed or water-limited conditions remain part of the overall SWP and VPD domain but do not influence the baseline regression, as they consistently fall below the upper limit. The inclusion of such data therefore does not compromise the definition of a unified baseline; rather, it reinforces its physiological meaning by clearly distinguishing non-limiting conditions from progressive soil water limitations across contrasting environments. The multi-site approach used in this study also allows bias reduction due to soil heterogeneity or transient hypoxia, which could affect studies carried out in specific areas.

### 4.3 Baseline range and interpretation of deviations as indicators of soil water limitation

It is noteworthy that the range of SWP values corresponding to the hazelnut baseline is relatively narrow (from approximately−0.28 to −0.67 MPa between VPD values of 1 and 5 kPa), while observed SWP values in the field extended to −1.4 MPa. This indicates that the baseline serves primarily as a stable upper boundary for plant water status, against which deviations can be interpreted as evidence of soil water limitation. The relatively limited baseline range compared to the broader physiological range observed in the orchard highlights its robustness as a reference line but also underscores the importance of monitoring actual SWP values over time.

### 4.4 Effect of grafting on plant water status and SWP baseline interpretation

The differences observed between grafted and own-rooted plants in terms of stomatal conductance, net photosynthesis, and, at specific times of the season, SWP, can be interpreted considering the distinct root system architecture associated with grafting onto *Corylus colurna*. Previous studies have demonstrated that grafted hazelnut trees develop deeper and more structured root systems compared to own-rooted plants, enabling access to deeper and more stable soil water reserves and promoting different water uptake strategies (Portarena et al., 2022; Vinci et al., 2023).

Portarena et al. (2022) showed that grafted hazelnut plants exhibit distinct carbon allocation patterns and deeper water uptake compared to own-rooted trees, resulting in differences in whole-plant hydraulic functioning and physiological regulation. Such traits may enhance the buffering capacity of grafted plants against short-term fluctuations in atmospheric demand, particularly under conditions of high VPD. In this context, a deeper rooting depth may allow grafted plants to sustain relatively high stomatal conductance and radiation interception during the early and mid-season phases without inducing more negative SWP values.

The effect of grafting is therefore relevant not only for plant physiological performance but also for irrigation-related parameters, including crop coefficients and the interpretation of plant-based water status indicators. Vinci et al. (2023) reported lower midseason crop coefficient values for grafted hazelnut orchards compared to those commonly reported for own-rooted systems, despite higher planting densities. This suggests a greater efficiency in water use and a modified relationship between canopy development, radiation interception, and transpiration in grafted trees.

In the present study, no statistically significant differences were detected in the SWP–VPD relationship between grafted and own-rooted plants, supporting the use of a single non-water-stressed baseline for both materials under well-watered conditions. However, the distinct rooting depth and water uptake dynamics associated with grafting may contribute to subtle differences in plant hydraulic regulation that are not fully captured under non-limiting soil moisture conditions. Under more heterogeneous soil water availability or during prolonged periods of high evaporative demand, these differences could potentially influence the stability or slope of the SWP baseline. Consequently, grafting should be considered a potential source of variability when applying plant-based irrigation indicators, and further targeted studies are required to assess its role in shaping baseline relationships under contrasting soil water regimes.

### 4.5 Stomatal regulation, isohydric behavior, and physiological limitations in hazelnut

Regarding the relation between SWP and *g*_s_, the relation showed a decline with decreasing SWP, with values in optimal conditions of approximately 0.2–0.6 mmol m^-^² s^-^¹ at approximately −0.4 MPa and approaching complete stomatal closure at about −1.4 MPa. The narrow range of optimal stomata conductance suggests the high susceptibility of filbert to heat stress and its tendency to have better performance in well-irrigated conditions. Compared to olive tree and *Prunus*, complete stomata closure happens respectively at −7 and −3/−2.5 MPa, more negative pressure than hazelnut (−1.4 MPa), confirming the conservative hydraulic strategy of this species. This behavior is consistent with the isohydric or “water-saving” strategy described for hazelnut, whereby stomatal closure is rapidly induced to preserve xylem integrity and avoid excessive declines in plant water potential (Marsal et al., 1997; Pasqualotto et al., 2018; Altieri et al., 2024).

Recent evidence further supports this interpretation by highlighting the high physiological sensitivity of hazelnut to water stress, which is associated with limited stomatal regulation capacity and a typically shallow root system (Rius-Garcia et al., 2025). The same study also reported significant genotypic differences, with cultivars such as “Tonda di Giffoni” showing greater ionic homeostasis and photosynthetic resilience under stress compared to more sensitive genotypes like “Tonda Gentile Romana”, reinforcing the concept that isohydric behavior in hazelnut is modulated by cultivar-specific traits rather than being a uniform species-level response.

Within this physiological framework, the steeper SWP–VPD relationship observed in hazelnut reflects a strong sensitivity to atmospheric demand rather than to soil water availability. Recent studies indicate that this response is driven by early stomatal closure associated with limited stomatal regulation capacity and a relatively shallow root system, resulting in rapid physiological downregulation even when soil water is not limiting (Cincera et al., 2019; Altieri et al., 2024; Rius-Garcia et al., 2025).

The low explanatory power of the *g*_s_–SWP relationship observed in this study is consistent with this isohydric behavior, as stomatal conductance in hazelnut is rapidly downregulated even under mild water stress to maintain relatively stable SWP (McCauley et al., 2024). Under these conditions, large variations in *g*_s_ can occur within a narrow SWP range, resulting in weak statistical coupling between the two variables and reinforcing the role of atmospheric demand as the primary physiological constraint. Interestingly, as shown in **Figure 3**, the observed decline of *g*_s_ at relatively high SWP values (−0.3 to −0.2 MPa) may indicate a possible limitation caused by transient waterlogging conditions. Similar short-term reductions in stomatal conductance under waterlogged conditions have been reported in other woody species, where hypoxic stress at the root level can rapidly constrain gas exchange (Levinsson et al., 2024).

### 4.6 Integration of plant-based water status indicators and irrigation cutoff thresholds into irrigation decision frameworks

Recent studies have emphasized the value of integrating plant-based water status indicators into advanced irrigation decision-making frameworks. In this context, diagnostic indices such as deviations from a reference baseline or the Crop Water Stress Index (CWSI) provide a physiological interpretation of crop water status that is directly linked to stomatal regulation and transpiration.

For hazelnut, McCauley et al. (2024) recently established a CWSI framework showing that SWP values above approximately −0.6 MPa correspond to low CWSI values (<0.2), representative of an unstressed physiological condition associated with stomatal conductance thresholds of approximately 0.2 mmol m^-2^ s^-1^. These findings are consistent with the SWP ranges identified in the present study as indicative of non-limiting soil water conditions and active stomatal function.

In addition to their diagnostic value, plant-based water status indicators can be directly linked to irrigation cutoff (ICO) strategies aimed at improving water productivity without compromising yield. Ortega-Farias et al. (2020) demonstrated that, for the cultivar “Tonda di Giffoni”, maintaining midday SWP at approximately −1.0 MPa allowed irrigation water savings of approximately 19% without significant reductions in stomatal conductance, net assimilation rate, or kernel weight. Conversely, when SWP declined to more negative thresholds (−1.3 to −1.6 MPa), marked reductions in gas exchange and kernel weight were observed. These results support the interpretation of deviations below the SWP baseline as indicators of soil water limitation and identify −1.0 MPa as a physiologically safe lower boundary for regulated deficit irrigation, whereas larger deviations should be regarded as entering a water stress zone associated with functional and productive penalties.

Beyond their diagnostic role, plant-based indicators such as SWP baselines and CWSI can also be integrated into multi-objective irrigation frameworks that aim to jointly optimize yield, quality, and water productivity under limited water availability. Indeed, the SWP baseline can support irrigation scheduling by identifying whether the irrigation volume applied per shift is excessive and should be divided into smaller, more frequent applications. Furthermore, it can provide an indication of the irrigation system’s efficiency in replenishing the water lost through transpiration, as estimated by the water budget method. Recent optimization-based approaches have demonstrated how crop water status or response functions can act as constraints or objective variables within such models. Within this perspective, the SWP baseline proposed here represents a robust physiological reference that can support both threshold-based irrigation scheduling—such as ICO strategies—and its integration into more advanced optimization-driven decision-support systems.

## 5 Conclusion

This study provides the first definition of an SWP baseline for filbert under non-limiting soil water conditions, developed across different environments and cultivars. The baseline, obtained from a large and heterogeneous dataset, showed a stable and linear relationship with VPD, since atmospheric demand is the principal driver of SWP when irrigation fully meets plant water requirements. The narrow SWP range defining the baseline, compared with the much wider range observed in field conditions, highlights its robustness as an upper physiological limit and reinforces its usefulness as a diagnostic tool for irrigation management.

Although the SWP–VPD baseline of filbert was less steep than that reported for other woody crops such as olive and *Prunus* spp., it provides a robust reference for interpreting plant water status under non-limiting soil moisture conditions. The pronounced sensitivity of filbert to water stress is instead reflected in the rapid decline of stomatal conductance at relatively high SWP values, supporting its classification as an isohydric species and highlighting its dependence on adequate soil water availability to sustain optimal gas-exchange performance. Furthermore, the observed reduction in stomatal conductance at high SWP values suggests that transient waterlogging may also impair plant functioning, emphasizing the need to avoid both water deficit and excessive irrigation. Overall, the SWP baseline developed in this work represents a practical and physiologically grounded reference for irrigation scheduling in hazelnut orchards. Using this baseline, growers can better identify conditions in which soil water availability is optimal and avoid unnecessary irrigation inputs that may reduce root efficiency or induce waterlogging stress. Future research should expand the dataset across additional cultivars, training systems, rootstock, and soil types, and evaluate the integration of SWP baseline monitoring with real-time irrigation decision tools. Such developments would enhance the sustainability and resilience of hazelnut production under increasingly variable climatic conditions.

## Data Availability

The datasets generated and/or analyzed during the current study are not publicly available because they are part of ongoing research activities, but are available from the corresponding author upon reasonable request.
