# Supplementary material for: Establishing hazelnut stem water potential baseline to improve water management
Source: Front Plant Sci. 2026 Mar 27;17:1771736. doi: 10.3389/fpls.2026.1771736 (PMC13065684; doi:10.3389/fpls.2026.1771736)
Supplement: Supplementary file 1 [file SupplementaryFile1.docx]

Supplementary Materials

Tabulated values of the hazelnut stem water potential baseline


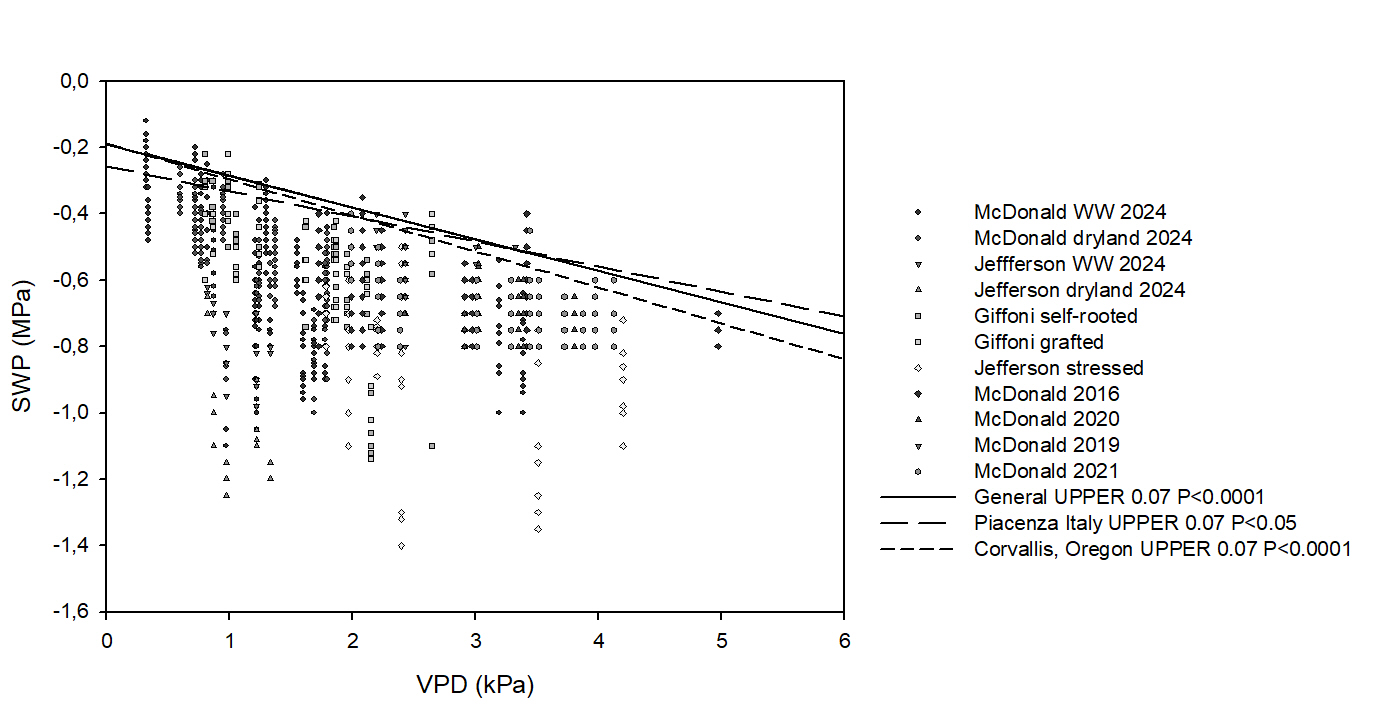


Fig S1. Relationship between midday stem water potential (SWP) and vapor pressure deficit (VPD) calculated using the upper 0.07 fraction of SWP values. Baseline regressions are shown for the pooled dataset (“General”) and calculated separately for the two experimental sites (Piacenza, Italy and Corvallis, Oregon). The similarity in slope and intercept across sites supports the robustness of the pooled SWP–VPD baseline presented in Figure 1.
